# Impact of Anemia Management on Bleeding Outcomes in Anticoagulated Patients: A Retrospective Cohort Analysis

**DOI:** 10.46989/001c.138102

**Published:** 2025-05-27

**Authors:** Shea-Lee Godin, Christopher Hanna, Edgar Naut, Sudhanshu Mulay

**Affiliations:** 1 Department of Medicine, University of Connecticut Health, Farmington, Connecticut, USA; 2 Department of Medicine, St. Francis Hospital, Hartford, Connecticut, USA; 3 Department of Hematology-Oncology, St. Francis Hospital, Hartford, Connecticut, USA

**Keywords:** anemia, anticoagulation, bleeding risk, bleeding severity, patient safety

## Abstract

Anticoagulation therapy is essential to manage thromboembolic conditions such as atrial fibrillation and venous thromboembolism. While effective, it carries significant bleeding risks, with annual rates ranging from 10–17% for all events and 2–5% for major bleeding. Anemia is an independent risk factor for anticoagulation-associated bleeding; however, guidelines lack recommendations for anemia screening and management before initiation.

In a retrospective analysis of 170 anticoagulated patients (mean age 63.7; 96 males, 74 females), 51.2% had baseline anemia. Anemia severity was significantly associated with bleeding events (χ²=15.7, p=0.003). Multivariate analysis confirmed that moderate (aOR=0.26, 95% CI:0.08–0.82, p=0.021) and no anemia (aOR=0.42, 95% CI:0.22–0.82, p=0.011) were associated with lower bleeding risk than mild anemia, while severe anemia remained uninterpretable due to small sample size. Patients aged ≥65 had higher bleeding risk (OR=2.8, 95% CI:1.5–5.1, p<0.01), though this did not reach significance in multivariate analysis (aOR=1.80, 95% CI:0.95–3.41, p=0.073). Multivariate analysis confirmed higher bleeding risks for warfarin (aOR=4.13, 95% CI:1.91–8.96, p<0.001) and rivaroxaban (aOR=3.67, 95% CI:1.69–7.97, p=0.001) compared to apixaban.

Our study found an association between anemia and bleeding events, though severe anemia did not correlate with bleeding, possibly due to small sample size. Direct oral anticoagulants like apixaban and rivaroxaban present lower bleeding risks than warfarin. Given anemia’s role in bleeding risk, we recommend routine screening before initiating anticoagulation to improve patient safety. Early assessment may help reduce bleeding complications, particularly in high-risk populations. Future studies should focus on multi-center trials to validate these findings and explore anemia subtypes.

## Introduction

*Anticoagulation* is a common medical intervention used for primary and secondary prevention of thromboembolic complications of vascular disease.^1,2^ Anticoagulation use is increasing; several clinical trials show its effectiveness for primary and secondary prevention of thromboembolic complications in atrial fibrillation (AFib), prosthetic heart valves, cardioembolic ischemic cerebral disease, myocardial infarction, and venous thromboembolism (VTE).^2^

Although there are substantial benefits to anticoagulant treatment, bleeding complications are not uncommon and can be life-threatening.^2,3^ These limit therapeutic benefit of anticoagulation.^2^ The literature suggests that the incidence of bleeding complications associated with therapeutic dosing of oral anticoagulant therapy varies from 10 to 17 per 100 patient-years for all bleeding complications, and from 2 to 5 per 100 patient-years for major bleeding complications.^2^

Over recent years, anemia has been established as an independent risk factor for significant bleeding during anticoagulation therapy, and as an independent predictor of overall mortality in patients with acute VTE.^4^ Severe anemia (hematocrit ≤30%) unrelated to acute bleeding has been associated with significant anticoagulant-related bleeding in inpatient and outpatient settings.^5,6^

Most bleeding risk scores now include anemia.^4^ Patients with anemia have a twofold higher risk of significant bleeding and a threefold higher risk of fatal bleeding than those without anemia on anticoagulation therapy.^4^ This risk is highest during the first year.^5,6^ It is similar when adjusted for potential confounders, such as cancer versus non-cancer patients.^4^

The literature indicates that anemia is a significant risk factor for significant and even fatal bleeding while on anticoagulation. Despite these findings, guidelines lack recommendations for routine baseline screening and management of anemia prior to anticoagulation initiation, as well as for routine screenings during therapy, particularly during the first year when bleeding risk is the highest.

This project evaluated the association between anticoagulation use and anemia, focusing on baseline hemoglobin levels, anemia management, monitoring practices, measures taken if anemia was present prior to initiation, and bleeding events. We aimed to determine if improved anemia monitoring and management practices before anticoagulation initiation could impact bleeding complications in patients undergoing anticoagulation therapy. The objectives included evaluating the relationship between baseline anemia severity and the incidence of bleeding events in anticoagulated patients, and identifying key comorbidities and other factors influencing the effectiveness of anemia monitoring and management.

## Methods

This retrospective study analyzed de-identified electronic health record (EHR) data from patients at a resident safety-net clinic in an urban city-center hospital. The hospital’s Institutional Review Board (IRB) reviewed and approved the protocol and complied with ethical guidelines for using de-identified patient data.

The sample size was determined to ensure adequate statistical power to detect meaningful associations between anticoagulation therapy, anemia severity, and bleeding risk. In a clinic population of 2,037 patients, our sample consisted of 170, providing a power of 85%. Descriptive statistics were used to explore these relationships. Patients were selected from the resident safety-net clinic’s population using the EHR system. Patients on long-term anticoagulation for AFib, VTE, or mechanical heart valves were included. Those on short-term prophylaxis during inpatient stays were excluded. Additionally, patients with inherited bleeding disorders (i.e. Von Willebrand’s disease or Hemophilia) and cirrhosis were excluded due to increased bleeding risk and coagulopathy.

SG and CH collected de-identified data over two weeks, focusing on demographics, baseline hemoglobin, anticoagulation details, and bleeding events by severity. Descriptive statistical methods were applied to explore the relationships between bleeding risk and the impact of different anticoagulants on patients with various severities of pre-existing anemia. The analysis was conducted according to the protocol, and all authors contributed to interpreting the findings. No randomization or clinical trial registration was involved.

A multivariate logistic regression analysis assessed the association between bleeding events and key clinical variables, including gender, anemia status, anticoagulant type, and age. Adjusted odds ratios (aOR) and 95% confidence intervals (CI) were calculated to quantify the independent effects of these variables on bleeding risk. Statistical significance was determined using a p-value threshold of <0.05, and all analyses were conducted using Python 3.13.

## Results

### Demographics (Table 1)

#### Gender and Age

A total of 170 patients were included in the analysis, comprising 96 males (56.5%) and 74 females (43.5%). Chi-square analysis for gender distribution showed no significant difference in proportions between males and females (χ²=3.09, p=0.08).

The mean age was 63.7 years (95% CI:61.5–65.9), with a standard deviation of 12.5 years, ranging from 31 to 88 years. The 25th percentile was 57.3 years, the median was 64 years, and the 75th percentile was 72 years. When broken down by gender, the mean age for males was 63.1 years with standard deviation of 10.8 years, and for females, it was 64.5 years with standard deviation of 12.5 years. An independent t-test comparing the mean ages revealed no significant difference by gender (t=1.01, p=0.31).

**Table 1.** Demographics

	Count (N=170)
**Gender**	
Male	96
Female	74
**Age Statistics**	
Mean Age (Years) ± SD	63.7 ± 12.5
95% CI (Age)	61.5 - 65.9
**Age Range**	
< 40	3 9
40–59	46
60–79	60
> 80	25
**Anticoagulant Type**	
Apixaban	73
Rivaroxaban	46
Warfarin	43
Other Anticoagulants	8
**Race/Ethnicity**	
White	25
Black	83
Hispanic	57
Asian/Other	5
**Anemia Severity**	
No Anemia	83
Mild Anemia	58
Moderate Anemia	15
Severe Anemia	2
**Bleeding Events**	
Yes	70
No	100
**Bleeding Type**	
Major Bleeding	14
Minor Bleeding	47

#### Race

The study population includes individuals from four racial categories: White (25 participants, 15.6%), Black (83 participants, 51.9%), Hispanic (57 participants, 35.6%), and Other Races (5 participants, 3.1%, including Asian/Unknown categories). Among White individuals, 56% were male and 44% were female. Black participants included 59.04% males and 40.96% females. Hispanic individuals comprised 55.77% males and 44.23% females, while participants from Other Races had equal gender distribution (40% male, 60% female). The age distribution varied across racial groups. Both males and females were evenly distributed across all age categories in each race.

**Figure 1.**
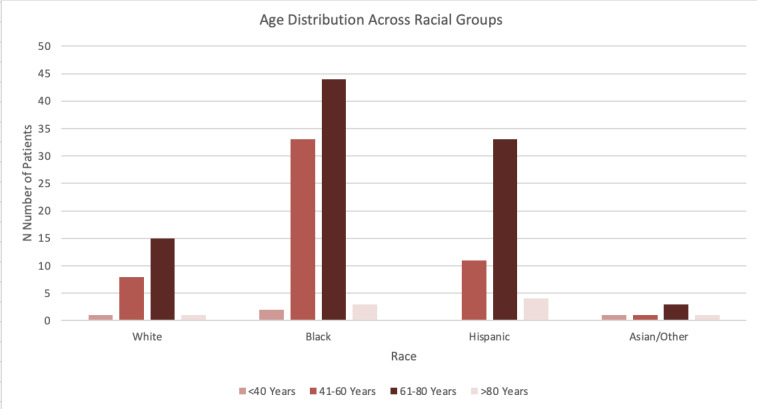
Age Distribution Across Racial Groups A bar chart illustrating the distribution of patient ages across different racial groups. The x-axis represents racial categories, including White, Black, Hispanic, and Other. The y-axis indicates the number of patients within each age range. Each racial group is subdivided into four age brackets: 18–40 years, 41–60 years, 61–80 years, and over 80 years. The bars are color-coded for clarity, showing trends in age distribution among racial demographics

Chi-square analyses showed no significant associations between race and age group (χ²=12.55, p=0.184) or race and gender distribution (χ²=0.78, p=0.855), indicating similar age and gender distributions across racial groups. However, a chi-square analysis of overall racial representation revealed significant differences (χ²=49.33, p<0.001), showing the distribution of racial categories were not uniform. Figure 1.

### Anemia Severity and Investigation

When evaluating anemia severity at the initiation of anticoagulation, 83 patients had normal hemoglobin. This group included 46 males and 37 females, with an age distribution of 22 patients under 40, 18 between 40 and 59, 30 between 60 and 79, and 13 aged 80 or older.

Among patients with mild anemia, 34.8% experienced bleeding (95% CI:26.2%–43.4%). A chi-square analysis confirmed a significant relationship between anemia severity and bleeding events (χ²=15.7, p=0.003). Moderate anemia was observed in 15 patients (17.2%), and severe anemia was rare, affecting only two (2.3%). Interestingly, no bleeding events occurred among patients with severe anemia. Anemia severity remained unknown for 12 patients.

Eighty-seven patients (51.2%) had defined anemia severity at initiation, including 58 patients (66.6%) with mild anemia. The latter, characterized by hemoglobin levels greater than 10 g/dL but below the normal range for age and gender, was more common among females (33) than males (25). By age, mild anemia was observed in 12 patients under 40, 19 aged 40–59, 18 aged 60–79, and nine aged 80 or older.

Moderate anemia, defined by hemoglobin levels between 8–10 g/dL, was present in 15 patients (17.2%), distributed evenly across genders (eight males and seven females). By age, moderate anemia was seen in two patients under 40, five patients aged 40–59, six aged 60–79, and two aged 80 or older. Severe anemia, defined by hemoglobin levels below 8 g/dL, was identified in just two patients (2.3%). Both involved males aged 60–79 years.

The severity of anemia in twelve patients was unknown; no hemoglobin or anemia workup data were available. This group consisted of seven males and five females, distributed across age groups: three under 40 years, four aged 40–59, four aged 60–79, and one aged 80 or older.

A significant proportion of the cohort (121 patients, 71.12%) had no anemia workup completed. This group included 68 males and 53 females, with age distributions of 25 patients under 40, 31 aged 40–59, 43 aged 60–79, and 22 aged 80 or older. Conversely, 32 patients (13.82%) underwent anemia investigations, including diagnostic testing and evaluations for potential underlying causes. This group comprised 18 males and 14 females, with seven patients under 40, nine aged 40–59, 10 aged 60–79, and six aged 80 or older.

Additionally, five patients (2.94%) were recommended for outpatient follow-up to investigate underlying causes of anemia. This group consisted of three males and two females, with one patient under 40, two aged 40–59, one aged 60–79, and one aged 80 or older.

### Anticoagulation Utilization

Among the 170 patients, the most initiated anticoagulant was apixaban, used by 73 (42.94%). Rivaroxaban followed this in 46 patients (27.06%) and warfarin in 43 (25.29%). Other anticoagulants were less frequently used, including enoxaparin sodium in three patients (1.76%), fondaparinux in one(0.59%), dabigatran etexilate in two (1.18%), and other agents in two (1.18%).

#### Use by Gender

Anticoagulant usage showed distinct patterns by gender. Apixaban was prescribed more frequently to females (42) than males (31), accounting for 57.5% of all anticoagulant use in females and 45.6% in males. Conversely, rivaroxaban was used more by males (27) than females (19), representing 29.2% of male anticoagulant use and 26% of female usage. Warfarin was nearly evenly distributed between genders, with slightly more males (24) than females (19) using this anticoagulant, accounting for 26.1% of male and 26% of female usage.

Enoxaparin use was evenly distributed, with two males and one female. Fondaparinux, dabigatran, and other agents were primarily prescribed to males (3). Chi-square analysis revealed no significant difference in anticoagulant use by gender (χ²=4.62, p=0.32).

#### Use by Age

Apixaban emerged as the most frequently used anticoagulant across all age groups, with peak usage observed in the 61–70 age range (24). In the <60 age group, apixaban (8) and rivaroxaban (7) were the most common choices. Among individuals aged 61–70, apixaban dominated (24), followed by rivaroxaban (11).

In the 71–80 age group, apixaban and rivaroxaban were used equally (18), with warfarin also seeing significant use (10). For patients aged 81 and older, apixaban remained the most common anticoagulant (23), followed by warfarin (14) and rivaroxaban (10). Warfarin was distributed relatively evenly but was slightly more prevalent among patients aged 81 and older. Enoxaparin, fondaparinux, dabigatran, and other agents were minimal and spread sparsely across all age groups. Chi-square analysis revealed significant preference for apixaban among older patients (χ²=28.7, p<0.001).

#### Use by Race

Apixaban was the most used anticoagulant across all racial groups, with most being Black (36). Rivaroxaban use was also concentrated among Black individuals (14), followed by smaller numbers of Hispanic (18) and White (6). Warfarin use was similarly highest among Black individuals (25), with Hispanic (12) and White (4) individuals comprising smaller proportions. Among other racial groups (e.g., Asian/Other), anticoagulant use was sparse, with apixaban being the predominant choice.

Chi-square test of independence showed no significant association between race and anticoagulant type (χ²=7.99, p=0.239), suggesting the observed differences in anticoagulant use across racial groups might be due to chance.

### Bleeding Events

Out of the total dataset, 70 patients (41.2%) experienced bleeding events while on anticoagulation, whereas 100 (58.8%) did not. A chi-square analysis comparing bleeding event rates across anticoagulants revealed significant differences (χ²=21.5, p<0.001), with warfarin showing the highest bleeding rate at 48.8%. Confidence intervals for bleeding events by anticoagulant type were as follows: apixaban (95% CI:13.6%–28.6%), rivaroxaban (95% CI:25.1%–47.5%), and warfarin (95% CI:36.1%–61.5%).

#### Types of Bleeding

A variety of bleeding events were documented, totaling 70 cases. Gastrointestinal bleeding (GIB) was the most frequently reported type, observed in 23 cases. Other types of bleeding included menorrhagia(3), epistaxis(2), hematuria(2), and hematomas(1). Less frequent bleeding types were hematemesis(1), intracranial hemorrhage(1), gingival bleeding(1), retroperitoneal bleeding(1), and hemoptysis(1). Additionally, nine cases involved multiple bleeding sites.

#### Bleeding by Gender

Bleeding events occurred slightly more frequently in males (40, 57.1%) compared to females (30, 42.9%). Among males, GIB was the most common type (12), followed by two instances each of epistaxis and hematomas and single cases of hematemesis, gingival bleeding, hemoptysis, and combined GIB with hematemesis. Five cases involving multiple bleeding sites were also reported.

In females, GIB was also the most frequently observed type (11), followed by three cases of menorrhagia, two cases of hematuria, and single cases of intracranial hemorrhage, retroperitoneal bleeding, and combined GIB with hemoptysis. Four cases involving multiple bleeding sites were documented. Despite these differences, a chi-square analysis showed no significant difference in bleeding rates between males and females (χ²=1.87, p=0.17).

#### Bleeding by Age

Bleeding events were significantly more frequent among patients aged 65 and older (χ²=10.2, p=0.0016). The distribution of bleeding events varied across age groups. Among patients under 40 years, there were eight cases, including three of menorrhagia, two of hematuria, and three of GIB. In the 40–59 age group, 18 bleeding events occurred, with GIB being the most common (7), followed by two instances each of hematomas and hemoptysis, and seven cases involving multiple bleeding sites. Patients aged 60–79 years experienced the highest number of bleeding events (35), including 15 cases of GIB, one retroperitoneal bleed, and 19 cases with multiple bleeding sites. Among patients aged 80 years and older, nine bleeding events were reported, predominantly GIB (6), along with three cases of hematemesis. This distribution highlights the increasing frequency and complexity of bleeding events with advancing age.

### Bleeding Severity

#### Definition of Major Bleeding

The International Society on Thrombosis and Haemostasis^7^ proposes the following criteria for major bleeding in non-surgical patients:

1. Fatal bleeding, and/or
2. Symptomatic bleeding in a critical area or organ, such as intracranial, intraspinal, intraocular, retroperitoneal, intra-articular, pericardial, or intramuscular with compartment syndrome, and/or
3. Bleeding resulting in a drop in hemoglobin level of 20 g/L^−1^(1.24 mmol L^−1^) or more, or leading to transfusion of two or more units of whole blood or red cells.

Minor bleeding constitutes all other bleeding events.

In the dataset, bleeding severity was categorized as follows: 47 cases (67.1%) as minor, 14 (20%) as major, and nine (12.9%) experiencing major and minor bleeding. Among the major bleeding cases, 12 patients exhibited symptomatic bleeding in a critical area or organ, and nine experienced a hemoglobin drop of 20 g/L^−1^ or more or required a transfusion of at least two units of blood. Notably, one case met the criteria for both fatal bleeding and bleeding in a critical area.

Regarding packed red blood cells administration, eight patients required one unit, seven required two units, and a smaller number required three or more units.

#### Bleeding Events by Anemia Severity

Distinct patterns emerged when analyzing bleeding events based on anemia severity at initiating anticoagulation. Among patients with mild anemia, 31 individuals (34.8%) experienced bleeding events, while 27 did not. Five (5.7%) patients with moderate anemia experienced bleeding, and 10 did not. Notably, no bleeding events were observed among the two patients with severe anemia. For those without defined anemia, 31 individuals (35.6%) experienced bleeding, whereas 52 did not.

When stratified by gender, females with mild anemia were slightly more likely to experience bleeding than males (20 versus 11). In the moderate anemia category, bleeding occurred slightly more often in males (3) compared to females (2). For patients without anemia, bleeding events were evenly distributed between genders, with 16 females and 15 males affected.

Age analysis highlighted patients aged 65 and older being disproportionately represented among those experiencing bleeding events, particularly in the mild and moderate anemia categories. Among patients with mild anemia, 23 of the 31 who experienced bleeding were aged 65 or older. Similarly, in the moderate anemia category, four out of five patients who experienced bleeding belonged to this age group. An age-adjusted logistic regression demonstrated that patients over 65 had a significantly higher risk of bleeding, regardless of anemia severity (Odds Ratio [OR]=2.8, 95% CI:1.5–5.1, p<0.01).

A post-hoc power analysis determined that at least 102 patients with severe anemia would be needed to detect significant difference in bleeding risk with 80% power. However, with only two patients in this category, the study was significantly underpowered to conclude the absence of bleeding events. The lack of observed bleeding is likely due to the small sample size rather than a true lack of association, highlighting the study’s limitations in assessing this relationship.

### Bleeding Events by Anticoagulation

Bleeding events varied by anticoagulant type. Among patients on apixaban, 15 experienced bleeding, while 53 did not, resulting in a bleeding rate of 22.06%. For rivaroxaban, 19 patients experienced bleeding, while 21 did not, yielding a higher bleeding rate of 47.5%. Warfarin had the highest proportion of bleeding, with 21 patients experiencing bleeding compared to 14 who did not, equating to a bleeding rate of 60.0%. Other anticoagulants were used less frequently and resulted in lower overall bleeding counts: enoxaparin was associated with two, while fondaparinux, dabigatran, and other agents each accounted for one or two cases of bleeding each.

Chi-square tests demonstrated significant association between anticoagulant type and bleeding events (χ^2^=13.44, p=0.009). Pairwise comparisons highlighted that warfarin users had significantly higher risk of bleeding compared to apixaban (OR=3.4, 95% CI:1.6–7.2, p=0.002). The bleeding rate for rivaroxaban was also significantly higher than for apixaban (OR=2.7, 95% CI:1.2–6.1, p=0.014), but the difference between rivaroxaban and warfarin did not reach statistical significance.

**Figure 2.**
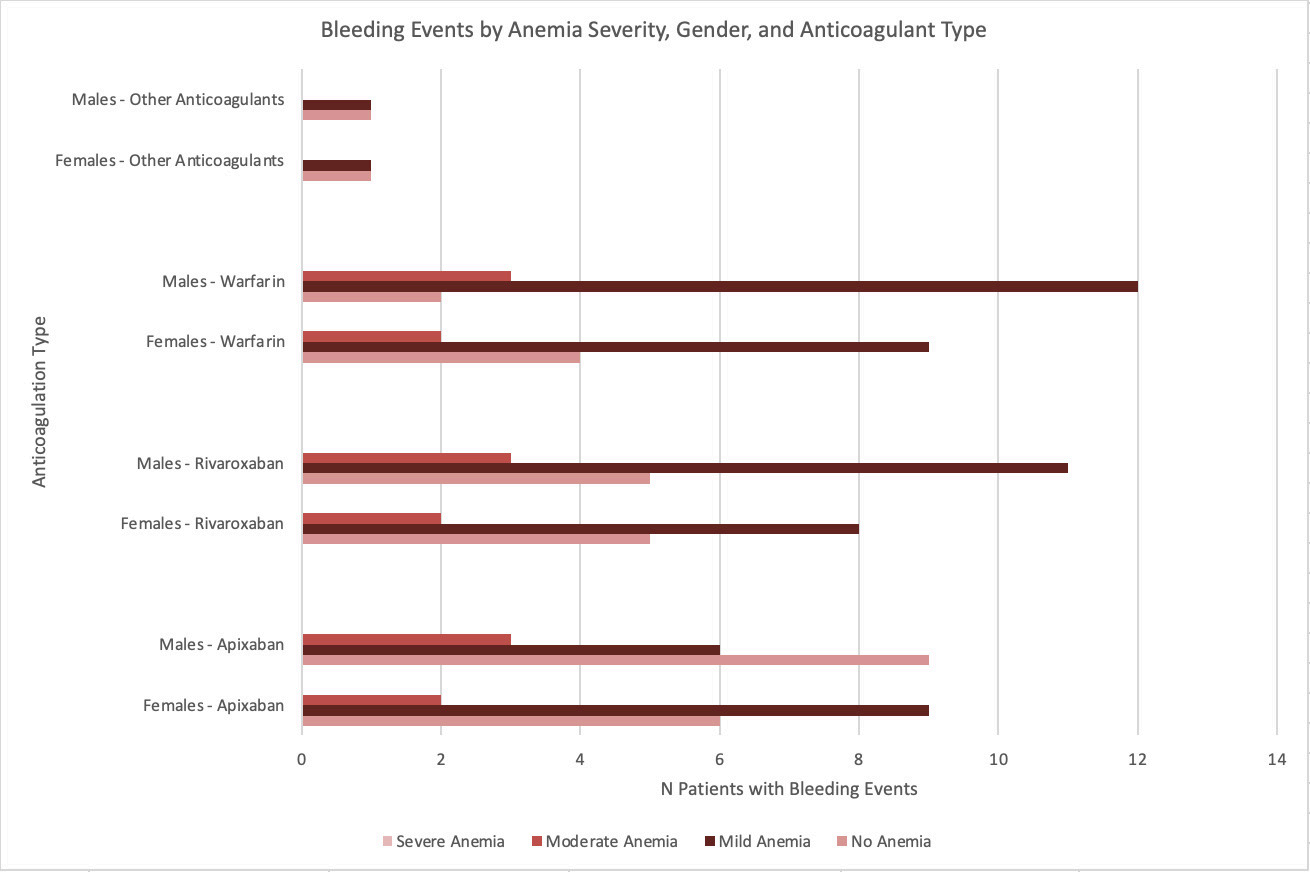
Bleeding Events by Anemia Severity, Gender, and Anticoagulant Type This figure shows the percentage of patients who experienced bleeding, stratified by anemia severity (No Anemia, Mild, Moderate, Severe), gender, and anticoagulant type. Each anemia severity group has separate bars for males and females, with stacked sections representing different anticoagulants (Apixaban, Rivaroxaban, Warfarin, Other). This visualization highlights how bleeding risk varies by anemia severity, gender, and anticoagulant selection.

Gender analysis revealed variations across anticoagulants. For apixaban, females experienced slightly more bleeding events than males (9 versus 6), while for rivaroxaban, males had a higher frequency of bleeding (11 versus 8). Similarly, for warfarin, 12 males experienced bleeding events compared to nine females. However, these gender differences were not significant(p>0.05). Figure 2 shows bleeding events by anemia severity and anticoagulation type regarding gender differentiation.

**Figure 3.**
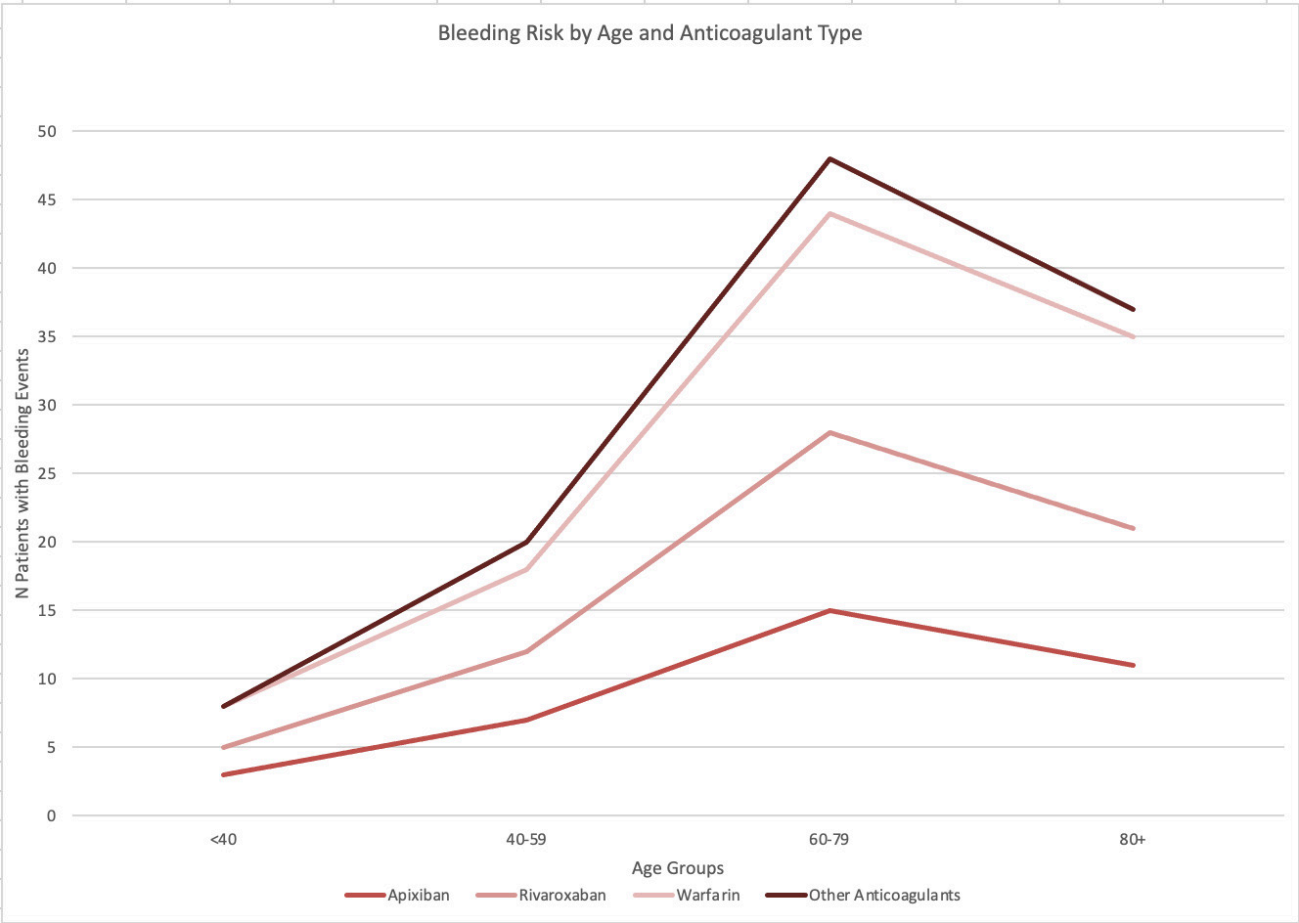
Bleeding Risk by Age and Anticoagulant Type This line graph illustrates the percentage of patients experiencing bleeding across different age groups (<40, 40–59, 60–79, 80+), categorized by anticoagulant type. Each line represents a different anticoagulant (Apixaban, Rivaroxaban, Warfarin, Other), showing how bleeding risk changes with increasing age.

Age analysis demonstrated bleeding events were more common in patients aged 65 and older across all anticoagulants. Among apixaban users who experienced bleeding, 11 of 15 cases involved patients aged 65 or older. For rivaroxaban, 13 of the 19 bleeding events occurred in this age group, while for warfarin, 16 out of 21 bleeding events involved patients aged 65 or older. Chi-square test confirmed a significant association between age (≥65 years versus <65 years) and bleeding events (χ^2^=7.41, p=0.0065). Figure 3.

#### Multivariate Analysis

Multivariate logistic regression analysis identified factors associated with bleeding events in anticoagulated patients, adjusting for gender, anemia status, anticoagulant type, and age. The baseline odds of bleeding were represented by the intercept (aOR=0.40, p=0.024), corresponding to a female patient <65 years with mild anemia on apixaban.

Moderate (aOR=0.26, 95% CI:0.08–0.82, p=0.021) and no anemia (aOR=0.42, 95% CI:0.22–0.82, p=0.011) were associated with lower bleeding risk than mild anemia. Severe anemia lacked interpretable results due to the small sample size.

Anticoagulation type significantly influenced bleeding risk.

Compared to apixaban, rivaroxaban (aOR=3.67, 95% CI:1.69–7.97, p=0.001), warfarin (aOR=4.13, 95% CI:1.91–8.96, p<0.001), and other anticoagulants (aOR=24.5, 95% CI:~4.4–137.3, p<0.001) had higher risks, though the last estimate was limited by the small sample size and wide confidence intervals.

Gender was not a significant predictor (aOR=1.13, 95% CI:0.60–2.16, p=0.71). Age ≥65 trended towards higher bleeding risk (aOR=1.80, 95% CI:0.95–3.41, p=0.073) but did not reach significance. The lack of significance may be attributed to sample size limitations or collinearity with other variables.

## Discussion

Anticoagulation is essential for treating many conditions, including VTE and AFib. However, it does not come without some risks, which should be identified prior to initiation.

### Comparison of Bleeding Risk Scores

Several bleeding risk scores have been developed, accounting for various factors. The most used ones are HAS-BLED and ORBIT, which have similar effectiveness as per Liu et al. (2023)^8,9^; others, including HEMORR2HAGES and ATRIA scores, are less commonly utilized in clinical practice due to their complexity and lower predictive performance compared to HAS-BLED and ORBIT.^10,11^

### Comparison of HAS-BLED and ORBIT Scores

HAS-BLED does not explicitly identify baseline hemoglobin in its calculation, but does consider age >65, hypertension, renal disease, liver disease, history of stroke or prior episodes of major bleeding “or predisposition” to bleeding, labile Internal Normalized Ratio (INR), medication predispositions, and alcohol use. Notably, HAS-BLED was developed initially with patients treated with warfarin in mind.

Conversely, ORBIT score *does* prompt physicians to determine if a patient has a baseline hemoglobin and hematocrit of <13g/dL and <40%, respectively, in males and <12g/dL and <36%, respectively, in females. Baseline anemia and prior bleeding give patients higher risk scores. ORBIT also considers age, renal disease, use of antiplatelet medications and gender.

Given that both scores consider age as a risk factor and that the primary population beginning anticoagulation therapy is older age >65, it could be considered a confounder in the risk of bleeding on anticoagulation. Tanaka et al. (2022) found moderate-severe anemia independently predicted mortality in patients >75 on apixaban but did not significantly increase the risk of bleeding events, strokes, or systemic emboli.^8^

### Brief Comparison of HEMORR2HAGES and ATRIA Scores

The ATRIA and HEMORR2HAGES scores assess bleeding risk in AFib patients on anticoagulation, but they differ in their complexity and predictive performance, making them less commonly utilized in clinical practice than HAS-BLED and ORBIT.^10^

The ATRIA score considers anemia, severe renal disease, age ≥75 years, prior hemorrhage, and hypertension. It assigns higher risk scores to patients with these conditions, particularly emphasizing anemia and severe renal disease.^10,11^ However, one study demonstrated that the ATRIA score had a c-index of 0.50 for predicting clinically relevant bleeding, indicating a lower, but modest predictive ability.^10^

Comparatively, the HEMORR2HAGES score incorporates a broader range of factors, including hepatic or renal disease, alcohol abuse, malignancy, advanced age, thrombocytopenia, hypertension, anemia, genetic factors, fall risk, and stroke.^10,11^ Despite its comprehensiveness, HEMORR2HAGES is more complex and has a lower predictive performance than HAS-BLED, with a c-index of 0.55.

### Anemia and Bleeding on Anticoagulation

Our study identified a significant association between anemia and bleeding events. This aligns with Goto et al. (2021), who reported that anemic patients had significantly higher risk of all-cause mortality and major bleeding after two-year follow-up.^12^ Other studies have also demonstrated low baseline hemoglobin being an independent predictor of all-cause mortality, cardiovascular events, and major bleeding in stable coronary artery disease patients.^13^ These studies highlight anemia as a critical risk factor for adverse outcomes on anticoagulation therapy.

### Severe Anemia and Lack of Bleeding Events

In our study, the two patients with severe baseline anemia did not experience bleeding events. This is likely due to small sample size and cautious patient selection, as clinicians consider patients with severe baseline anemia to be at high risk when starting anticoagulation. In these cases, benefits must significantly outweigh the risks of initiating anticoagulation. However, despite careful patient selection, studies consistently show that all-cause mortality and major bleeding are significantly higher in patients with severe anemia than in those with mild or moderate anemia.^12^

#### Restarting Anticoagulation After Bleeding Events

Interestingly, a meta-analysis of seven retrospective cohort studies found that restarting anticoagulation after a bleeding event reduced the risk of recurrent VTE without significantly increasing bleeding risk, ultimately lowering all-cause mortality.^14^ In this case, the cause of anemia is known before (re)-starting anticoagulation. Our study supports the routine evaluation of anemia before initiating anticoagulation, regardless of whether a prior bleeding event has occurred.

#### Drug Class Comparisons

Direct oral anticoagulants (DOACs), such as apixaban and rivaroxaban, have largely replaced Vitamin K Antagonists (VKAs) like warfarin for VTE management. This is primarily due to the higher bleeding risk associated with VKAs, particularly in patients with a HAS-BLED score ≥3, compared to DOACs.^10^ Numerous studies have demonstrated a lower bleeding risk in DOACs compared to VKAs.^15,16^ However, VKAs remain preferred for patients with VTE related to triple-positive antiphospholipid syndrome, valvular AFib and mechanical valves.^17^

#### Racial Disparities in Anticoagulation Use

Tedla et al. (2020) identified racial disparities in anticoagulation, noting that racial minorities with AFib were less likely to receive DOACs than White patients, even after adjusting for socioeconomic factors.^17^ DOAC use was also associated with a lower risk of stroke and bleeding events compared to VKA.^18^

Our study population reflected an urban, underrepresented demographic. Non-Hispanic White patients comprised 15.6% of the population, significantly lower than the national (57.8%) and Connecticut (66.4%) averages. Black patients accounted for (51.9%), overrepresenting the national average (13.6%) and that of Connecticut (10.8%). Hispanic/Latino patients comprised 35.6%, exceeding the national average (18.9%) and closely aligning with Connecticut’s (17.3%). Asian and unknown race categories comprised 3.1% of the cohort.

## Limitations and Future Directions

One notable limitation is the small sample size of 170 patients from a single-center safety net resident clinic. Only one patient was excluded based on the criteria. While this approach ensured a comprehensive capture of eligible patients and maintained statistical power within the broader cohort, the small sample size may limit the ability to detect subtle effects. Though racially diverse and reflective of underserved communities, the study population may overrepresent certain demographics compared to state and national populations. However, the single-center focus on low-income, underinsured patients may limit generalizability to other healthcare environments with different socioeconomic profiles.

Social determinants of health may affect anemia management, anticoagulation adherence and potentially bleeding risk outcomes. These disparities are particularly relevant in safety-net settings where patients may face significant barriers to care, hindering timely and consistent management of their conditions. Larger, multi-center studies are needed to validate the findings and enhance their generalizability to diverse populations and healthcare settings.

Another limitation is the retrospective nature, which relies on EHR and can introduce selection bias, as only patients with complete EHR were included. Additionally, unknown omissions from medical records, such as minor bleeding events that resolved without intervention may not have been recorded, potentially leading to underestimating overall bleeding rates. Prospective studies are needed to capture more comprehensive data on bleeding events and associated risk factors.

Anemia was associated with increased bleeding severity, but subtypes, such as iron deficiency anemia and anemia of chronic disease, were not analyzed due to the lack of a systematic workup to determine the etiology of anemia in patients initiated on anticoagulation therapy. This limitation prevents a more precise analysis of the relationship between anemia subtypes and bleeding outcomes, which could have provided insights into which subtypes contribute most significantly to bleeding risks, especially in patients with comorbidities like chronic kidney disease. Future research should consider categorizing anemia subtypes to further explore this potential connection.

### Recommendations

We recommend the following clinical practice guidelines. All patients discharged from the hospital on anticoagulation with anemia at baseline (Hb <12.5 for females; Hb <13.5 for males) should be worked up for the etiology. This entails a complete blood count, iron panel and ferritin, B12 and folate, and reticulocyte count. If these initial tests do not elicit a clear cause, further exploration with haptoglobin, LDH, zinc, copper may be necessary. Suspected blood loss anemia warrants considered exploration with endoscopy and colonoscopy. Periodic monitoring in the first year is advised.

Proactively identifying and managing anemia prior to starting anticoagulation can improve patient outcomes by increasing anticoagulation safety and reducing the likelihood of bleeding complications. Addressing underlying conditions contributing to anemia via respective treatment options (e.g., iron supplementation, erythropoiesis-stimulating agents) could allow clinicians to minimize the association with worse bleeding outcomes.

**Table 2.** Bleeding Event Rates and Adjusted Bleeding Risk by Anemia Severity, Anticoagulant Type, and Other Risk Factors

Factor	Bleeding Event Rate (%)	Adjusted Odds Ratio (aOR), 95% CI)	p-value
**Anemia Severity**			
No Anemia	31/87 (35.6%)	1.0 (Reference)	—
Mild Anemia	31/58 (34.8%)	1.5 (1.1–2.2)	0.021
Moderate Anemia	5/15 (5.7%)	0.26 (0.08–0.82)	0.011
Severe Anemia	0/2 (0.0%)	Not calculated	—
**Anticoagulant Type**			
Apixaban	15/68 (22.1%)	1.0 (Reference)	—
Rivaroxaban	19/40 (47.5%)	3.67 (1.69–7.97)	0.001
Warfarin	21/35 (60.0%)	4.13 (1.91–8.96)	<0.001
Other Anticoagulants	6/27 (22.2%)	24.5 (4.4–137.3)	<0.001
**Other Factors**			
Age > 65	—	1.80 (0.95–3.41)	0.073
Male v. Female	—	1.13 (0.60–2.16)	0.71

This table presents both the observed bleeding event rates (percentage of patients who experienced bleeding) and the adjusted odds ratios (aOR) for bleeding risk after controlling for confounders such as age and gender. Bleeding event rates indicate the proportion of patients within each category who experienced a bleeding event, while adjusted odds ratios quantify the increased bleeding risk.

### Authors’ Contribution

Conceptualization: Shea-Lee Godin (Lead). Methodology: Shea-Lee Godin (Equal), Christopher Hanna (Equal). Formal Analysis: Shea-Lee Godin (Equal), Christopher Hanna (Equal). Investigation: Shea-Lee Godin (Equal), Christopher Hanna (Equal). Writing – original draft: Shea-Lee Godin (Equal), Christopher Hanna (Equal). Writing – review & editing: Shea-Lee Godin (Equal), Christopher Hanna (Equal), Sudhanshu Mulay (Equal). Funding acquisition: Shea-Lee Godin (Equal), Christopher Hanna (Equal), Sudhanshu Mulay (Equal). Project administration: Shea-Lee Godin (Lead). Visualization: Shea-Lee Godin (Equal), Christopher Hanna (Equal), Sudhanshu Mulay (Lead). Resources: Edgar Naut (Equal), Sudhanshu Mulay (Equal). Supervision: Edgar Naut (Equal), Sudhanshu Mulay (Equal).

### Competing of Interest – COPE

No competing interests were disclosed.

### Ethical Conduct Approval

This study was approved by the Institutional Review Board (IRB) of St. Francis Hospital and Medical Center (IRB # SFH-25-10). The research was conducted in accordance with the ethical standards outlined in the 1964 Declaration of Helsinki and its subsequent amendments or comparable ethical standards. The IRB determined that informed consent was not required for this study.

### Informed Consent Statement

All authors and institutions have confirmed this manuscript for publication.

### Data Availability Statement

Data supporting the findings of this study are available upon request. The de-identified patient data used for this retrospective analysis can be obtained by contacting Dr. Shea-Lee Godin at sgodin@uchc.edu. Access to the data will be granted following an appropriate review of the request. The data will be available for one-year post-publication in accordance with the IRB approval. The study protocol will be available indefinitely.
